# To be or not to be: the role of CD56 in multiple myeloma

**DOI:** 10.18632/oncotarget.28350

**Published:** 2023-01-26

**Authors:** Francesca Cottini, Don Benson

**Keywords:** myeloma, targeted therapies, CD56, signaling

Optimal management of Multiple Myeloma (MM), a disorder of clonal malignant plasmacells (PCs), is hampered by the lack of strategies to treat specific subsets of patients. Despite genetic, epigenetic, and mutational heterogeneity, every patient still receives similar regimens without accounting for this variability. This can lead to unsatisfactory or short-lasting responses especially in the relapsed settings or in patients with high-risk chromosomal abnormalities such as t(4;14), 1q21+, or deletion(17p) [1].

MM cells express on their surface a variety of antigens, which help to discern normal reactive PCs from malignant MM cells [2]. These antigens are also targets for monoclonal antibodies, bispecific engagers, or chimeric T/NK cellular therapy [3, 4]. Among them, there is CD56 or NCAM1 (Neuronal Cell adhesion molecule 1), a glycoprotein normally present in neurons, Natural Killer (NK), and T cells, but aberrantly expressed in more than seventy percent of patients with MM [5].

Writing in Molecular Cancer Research [6], Cottini et al., first analyzed the clone size of CD56-expressing clonal MM cells in a database of more than 700 hundred patients at diagnosis, showing a wide range among patients and an increase of clone size in the setting of disease progression. Historically, lack of CD56 has been associated with inferior outcomes, especially being a feature of primary plasma cell leukemia, an aggressive form of plasma cell dyscrasia [7]. However, patients with extramedullary disease or secondary plasma cell leukemia have persistence of CD56 expression, thus suggesting that the biology of these two diseases might be different [8]. Using a 10-percent clone size cutoff, the authors demonstrated inferior overall survival outcomes and shorter responses to autologous stem cell transplant in patients with more than 10 percent of CD56-expressing clonal cells. Moreover, the CD56 clone size and CD56 mRNA expression was greater in patients with t(4;14), a poor prognostic feature.

To determine CD56 phenotype and mechanism of action in MM, the authors modulated the expression of CD56 and its downstream targets. Overexpression of CD56 in negative MM cell lines promoted cell growth, and adhesion to bone marrow stromal cells, while the opposite occurred with CD56 silencing in CD56-positive cell lines. The surface expression of CD56 triggered the activation of the P90 ribosomal S6 kinase A3 (RSK2), and the phosphorylation of cAMP responsive element binding protein 1 (CREB1). Once phosphorylated, CREB1 activated transcription of antiapoptotic genes, such as *BCL2* and *MCL1*. The authors then utilized both genomic and pharmacological inhibition of RSK2 (BI-D1870 compound) and CREB1 (666-15 compound), showing that this approach specifically induces MM cell death in CD56-high MM patients (>10% of CD56-expressing clonal MM cells), while it is less effective in CD56-low MM patients.

Immunomodulatory drugs (IMIDs), such as lenalidomide, promote the degradation of proteins IKZF1/3 by binding to CRBN [9, 10]. Changes in CRBN expression by deletion, mutations, or downregulation have been linked to IMID resistance [11, 12]. The authors proved that cells overexpressing CD56 have lower levels of CRBN, the target of lenalidomide, implicating CD56 in the response to this drug. Conversely, CREB1/RSK2 blockade rescued CRBN levels in CD56-high MM cells and increased lenalidomide response. While CREB1 inhibitors are in pre-clinical development [13, 14], inhibitors of RSK2, such as PMD-026, are already in clinical trials for triple negative breast cancer, showing tolerable safety profiling [15]. Therefore, future studies are warranted to evaluate these inhibitors in patients with CD56-high MM.

**Figure 1:**
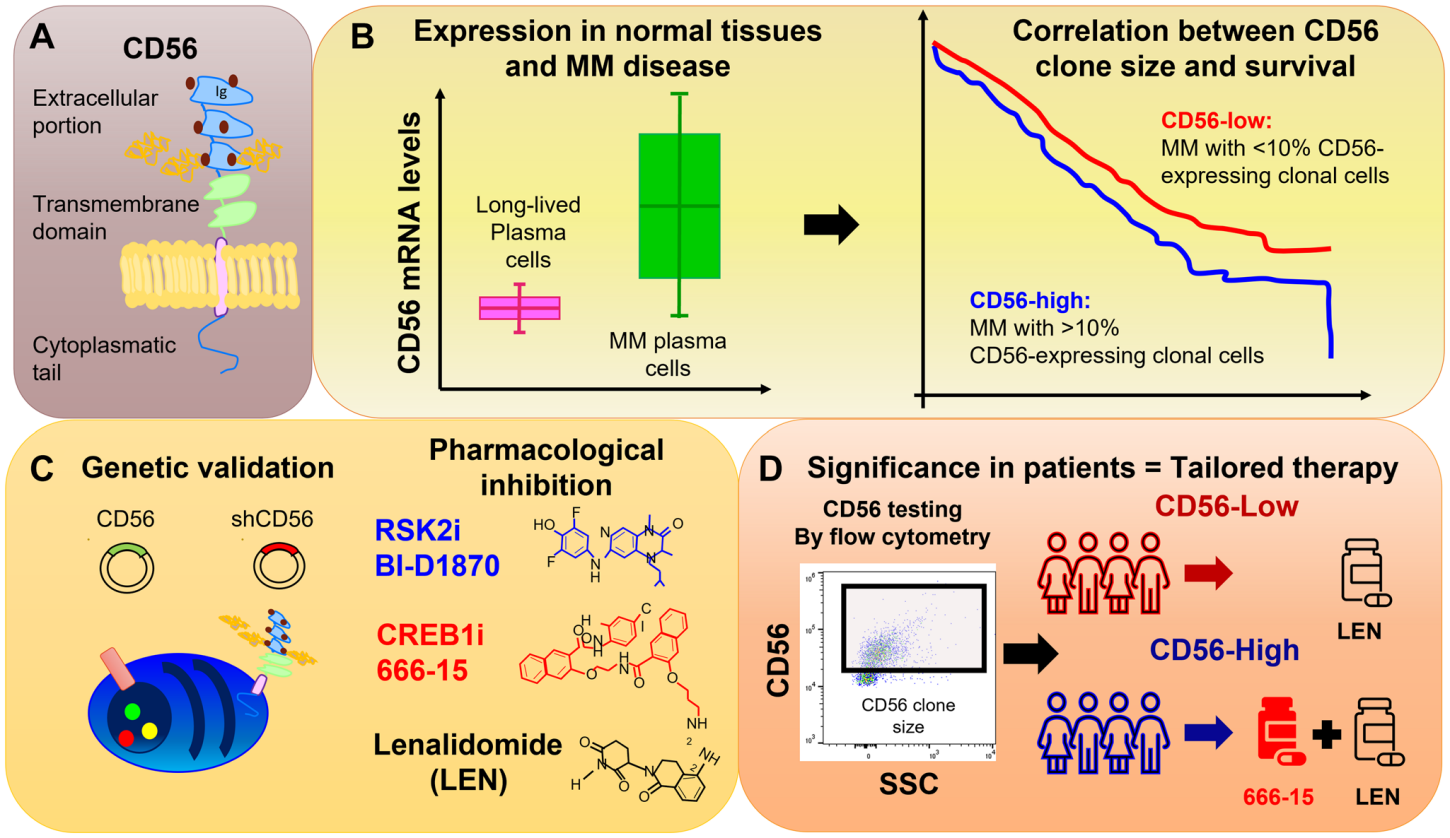
Graphical representation of the main findings of the summarized paper. (**A**) The most expressed CD56 isoform in MM creates a transmembrane protein with an extracellular portion and a cytoplasmatic tail. (**B**) Left. CD56 expression is greater in malignant MM cells compared with normal plasma cells. Right. The presence of more than 10% of clonal CD56-expressing cells (clone size) correlates with inferior outcomes. (**C**) CD56 modulation by overexpression or silencing (genetic validation) or treatment with experimental drugs (pharmacological inhibition), such as RSK2 inhibitor BI-D1870 (RSK2i), CREB1 inhibitor 666-15 (CREBi) or lenalidomide, affects MM survival. (**D**) CD56 testing by flow cytometry allows grouping patients based on CD56 clone size. CD56-high patients are more sensitive to RSK2i or CREB1i in combination with lenalidomide.

In summary, this study provides a detailed description of CD56 role in MM, opening new clinically relevant scenarios. The authors’ preclinical data support the use of synthetic lethal approaches by CREB1/RSK2 inhibition in combination with lenalidomide, as a strategy to overcome CRBN downregulation in CD56-high MM. CD56 expression is routinely tested on bone marrow aspirates by flow cytometry or on core biopsies by immunohistochemistry. Since the majority of clinical laboratories have the capability to perform CD56 staining and define a threshold of positivity, CD56 expression can be both a prognostic and predictive factor of response to therapies, an unmet need in the MM field (Figure 1).
